# Dr. Frank Kraft and the Cleveland College

**Published:** 1898-12

**Authors:** 


### DR. FRANK KRAFT AND THE CLEVELAND COLLEGE.

A request made by the students of the Senior Class of the Cleveland Homœopathic Medical College to the Faculty of that institution asking that Dr. Frank Kraft be restored to his old position of Professor of Materia Medica, and which request was refused, has been the cause of much discussion and feeling in the College.

Dr. Frank Kraft is one of the most prominent physicians in Cleveland, and he has a wide reputation as an able and progressive doctor. He is editor of *The American Homœopathist*, and he left a professorship of materia medica in the Medical Department of the University of Michigan several years ago to accept a similar chair in the old Cleveland Medical College. When that College was consolidated with the Cleveland University of Medicine and Surgery into the Cleveland Homœopathic College, Dr. Kraft was appointed to the chair of Materia Medica in the united institution. Dr. Kraft resigned his connection with the institution last summer. Dr. H. H. Baxter was then appointed to the chair of Materia Medica, which he still holds.

Dr. Kraft was personally very popular with the students as well as with a number of members of the Faculty. Recently the Seniors decided that they wanted Dr. Kraft to lecture to them on materia medica as well as Dr. Baxter, so they held a meeting and appointed a committee to wait on the Faculty and request that one hour a week be added to instruction on this branch, and that Dr. Kraft be appointed to take care of it. The Faculty considered the request presented by the committee, which consisted of H. J. Austin, A. J. Brainard, and A. W. Mercer, but refused it. It is claimed that Dr. Woods and Dr. Baxter, who are said to be leaders of the old Cleveland Medical College party in the Faculty, opposed the request, and that it was turned down despite the fact that a number of Faculty members of both factions favored it. However, the Faculty, to soothe the Seniors, added the additional hour to the curriculum, and selected Dr. Justin E. Rowland, Junior Professor of Materia Medica, to take charge of it.

Dr. Kraft bears the reputation of being one of the ablest and most advanced lecturers in that branch of medical science in the country. The Seniors say they recognized this fact, and without any disparagement of the ability of Dr. Baxter, deemed it to their interest to get the teachings of both.

H. J. Austin, who was chairman of the Senior's Committee, was visited last evening. He refused to discuss the matter in any manner beyond saying that Dr. Kraft would not be taken back into the Faculty as long as it was made up of its present elements. The above facts were learned from a number of the Seniors familiar with the matter, and from members of the Faculty.

Efforts were made to find Dr. W. E. Phillips and Dr. G. J. Jones, Dean and Vice-Dean respectively of the College, last night, but without success. Dr. Kraft was visited, and was told the situation. He expressed great surprise, and said it was all news to him.

"Things were made so unpleasant for me," said the doctor, "that last summer I was forced to resign from the Faculty. Dr. Woods was the leader of the opposition to me. That is all I care to say about the matter."

It is said tha Dr. Kraft's editorials in the medical journal of which he is editor, advocating purer teachings of homœopathic medicine, aroused considerable enmity toward him on the part of the older physicians on the Faculty. Another cause for opposition to Dr. Kraft, it is said, was his reading of a paper before the National Association of Physicians this year, advocating his advanced method of teaching Homœopathy by the aid of blackboard, charts, and pictures. Some of the editorials were construed by the dissatisfied ones to reflect on the Cleveland Homœopathic College.

Dr. Kraft denied this last evening. He declared he had always referred to homœopathic colleges in general.—*The News and Herald*, Cleveland, O., Nov. 11th, 1898.

